# Activation of Calcium-Activated Chloride Channels Suppresses Inherited Seizure Susceptibility in Genetically Epilepsy-Prone Rats

**DOI:** 10.3390/biomedicines10020449

**Published:** 2022-02-15

**Authors:** Miracle Thomas, Mark Simms, Prosper N’Gouemo

**Affiliations:** Department of Physiology and Biophysics, Howard University College of Medicine, Washington, DC 20059, USA; miracle.thomas@bison.howard.edu (M.T.); mark.simms@bison.howard.edu (M.S.)

**Keywords:** acoustically evoked seizures, EACT, generalized tonic-clonic seizures, inherited epilepsy, TMEM1A channels, wild running seizures

## Abstract

Inherited seizure susceptibility in genetically epilepsy-prone rats (GEPR-3s) is associated with increased voltage-gated calcium channel currents suggesting a massive calcium influx resulting in increased levels of intraneuronal calcium. Cytosolic calcium, in turn, activates many processes, including chloride channels, to restore normal membrane excitability and limit repetitive firing of the neurons. Here we used EACT and T16Ainh-A01, potent activator and inhibitor of calcium-activated channels transmembrane protein 16A (TMEM16A), respectively, to probe the role of these channels in the pathophysiology of acoustically evoked seizures in the GEPR-3s. We used adult male and female GEPR-3s. Acoustically evoked seizures consisted of wild running seizures (WRSs) that evolved into generalized tonic-clonic seizures (GTCSs) and eventually culminated into forelimb extension (partial tonic seizures). We found that acute EACT treatment at relatively higher tested doses significantly reduced the incidences of WRSs and GTCSs, and the seizure severity in male GEPR-3s. Furthermore, these antiseizure effects were associated with delayed seizure onset and reduced seizure duration. Interestingly, the inhibition of TMEM16A channels reversed EACT’s antiseizure effects on seizure latency and seizure duration. No notable antiseizure effects were observed in female GEPR-3s. Together, these findings suggest that activation of TMEM16A channels may represent a putative novel cellular mechanism for suppressing GTCSs.

## 1. Introduction

Epilepsy is one of the most common chronic neurological disorders characterized by recurrent seizures, which result from hypersynchronous discharges of neurons in specific brain networks. This disorder is associated with increased morbidity and death, and generalized tonic-clonic seizures (GTCSs) are the most common risk factor of sudden unexpected death in epilepsy, the leading cause of death in patients with epilepsy [1,2]. Significant progress has enhanced our understanding of the pathogenesis and pathophysiology of seizures and epilepsy, resulting in numerous antiseizure medications. However, some seizures are still refractory to optimal treatment with two or more antiseizure medications in about one-third of patients with epilepsy [3,4,5,6]. Therefore, there is an urgent need to develop additional therapeutic approaches based on new mechanisms underlying neuronal hyperexcitability that leads to seizures. Elevated levels of intraneuronal Ca^2+^ contributed to the generation and propagation of seizure activity and activated multiple Ca^2+^-dependent mechanisms, including Ca^2+^-activated chloride channels (CaCCs) [7,8,9,10]. Hence, systemic administration of inhibitors of voltage-gated Ca^2 +^ channels (VGCCs) and activator of small conductance Ca^2+^-activated K^+^ channels markedly suppressed acoustically evoked seizures in the genetically epilepsy-prone rats (GEPRs) and DBA/2 mice [11,12,13,14,15]. These findings suggested a functional and molecular remodeling of these channels, at least in the inferior colliculus (IC), the site for initiating acoustically evoked seizures in the GEPRs [11,12,13,14,15]. Accordingly, we found an upregulation of VGCCs and current density in the IC of the GEPR-3s, the moderated seizure severity strain of the GEPR [16,17]. Although a significant accumulation of intraneuronal chloride accompanies seizure activity, the role of CaCCs in the initiation and propagation of seizures has not been fully understood [18,19]. CaCCs are voltage-gated channels activated by rising intracellular Ca^2+^ concentration and play multiple physiological roles, including neuronal excitability regulation [20,21]. Anoctamin and bestrophin are the significant components of CaCCs; furthermore, anoctamins form a family of transmembrane (TMEM16) proteins, including TMEM16A, TMEM16B, and TMEM16C [22,23,24,25,26]. Interestingly, TMEM16A mRNA and proteins were expressed in auditory brainstem nuclei; such expression may also occur in the IC [27]. Here, we probe the role of TMEM16A channels in the mechanisms underlying seizures by evaluating the effects of EACT and T16Ainh-A01, potent activator and inhibitor of TMEM16A channels, respectively, on acoustically evoked seizure susceptibility in the GEPR-3, a model of inherited generalized tonic-clonic epilepsy.

## 2. Materials and Methods

### 2.1. Animals

The present study used 54 GEPR-3s (eight-week-old, male, and female) obtained from our animal colony maintained at Howard University College of Medicine. The GEPR-3s were housed in a temperature/humidity-controlled room on a 12 h/12 h light/dark cycle with free access to food and water. We made all possible efforts to minimize the number of animals used in experiments and their discomfort. Thus, the same animals were used as controls for each tested dosage of a given pharmacological agent. The Institutional Animal Care and Use Committee approved all experimental procedures (Protocol MED-20-04) following the National Institutes of Health Guide for the Care and Use of Laboratory Animals [28].

### 2.2. Acoustically Evoked Seizure Testing

Following administration of vehicle, EACT, and T16Ainh-A01, GEPR-3s were placed in an acoustic chamber (Med Associates, St. Albans, VT, USA) and tested for acoustically evoked susceptibility at 0.5 h, 1 h, and 2 h post-treatment. To evaluate the long-lasting effects of EACT and T16Ainh-A01, the GEPR-3s were again tested for acoustically evoked susceptibility 24 h later. To induce seizures, an acoustic stimulus that consisted of pure tones at a 100–105 decibels sound pressure level (Med Associated, St. Albans, VT, USA) was presented until either seizure was elicited, or 60 s passed with no seizure activity. The GEPR-3s were closely monitored following the administration of EACT and T16Ainh-A01. The phenotype of seizures was classified into seven stages [29]: stage 0, no seizures in response to an acoustic stimulus; stage 1, wild running seizures (WRSs); stage 2, two or more episodes of WRSs; stage 3, one episode of WRSs followed by generalized tonic-clonic seizures (GTCSs) characterized by tonic dorsiflexion of the neck, tonic flexion of shoulder and bouncing clonic seizures (or clonus, i.e., tonic-clonic seizures while the animal is lying on its belly); stage 4, two episodes of WRSs followed by GTCSs, stage 5, one episode of WRS followed by GTCSs and tonic forelimb extension (FLE, partial tonic seizures); and stage 6, two episodes of WRSs followed by GTCSs and FLE.

In another set of experiments, male and female GEPR-3s (*n* = 6/group) were used to assess the general behavior (up to 48 h) following administration of EACT at the dose of 2.5, 5, and 10 mg/kg body weight (p.o.) and T16Ainh-A01 at the dose of 10 mg/kg body weight (p.o.). We recorded the occurrence of lethargy, ataxia, tremor, Straub’s tail, change in body temperature, and spontaneous seizures. We humanely euthanized all animals at the end of the experiments.

### 2.3. Pharmacological Treatments

To evaluate the role of CaCCs in inherited seizure susceptibility in the GEPR-3, we used EACT (3,4,5-Trimethoxy-*N*-(2-methoxyethyl)-*N*-(4-phenyl-2-thiazolyl)benzamide) (R&D systems, Minneapolis, MN, USA), and T16Ainh-A01 (2-[(5-Ethyl-1,6-dihydro-4-methyl-6-oxo-2-pyrimidinyl)thio]-*N*-[4-(4-methoxyphenyl)-2 thiazolyl]acetamide (R&D Systems, Minneapolis, MN, USA), potent activator and inhibitor of TMEM16A channels, respectively. For EACT and T16Ainh-A01 experiments, the GEPR-3s were randomly separated into groups of *n* = 9 and *n* = 6, respectively, and were used as their controls. The GEPR-3s were first tested for acoustically evoked seizures 30 min following vehicle administration. Those GEPR-3s exhibiting seizures were referred to as controls and subsequently used for pharmacological studies one hour later. Eact (2.5, 5 and 10 mg/kg body weight) and T16Ainh-A01 (10 mg/kg body weight) were dissolved in dimethyl sulfonic acid (1%) and sterile water using sonication (80 kHz, 100% power); the solutions were filtered and administered 30 min before seizure testing. The vehicle, Eact, and T16Ainh-A01 were given per os (p.o.) by gastric intubation with a volume of 0.2 mL/100 g body weight using an 18-gauge stainless steel feeding needle (round tip, ball diameter 3 mm). The tested dose range and the 30 min timeframe interval were chosen based on our previously published in vivo pharmacological studies and preliminary data. The order of seizure testing was randomized and counterbalanced by dose and sex. For EACT experiments, each seizure testing dosage was performed at a minimum of 72 h to allow its washout.

### 2.4. Data Analysis

The investigators were blinded to group allocation during experiments and data analysis. The Origin 2021 software (Origin Northampton, MA, USA) and Primer 6th edition software (Primer of Biostatistics, McGraw-Hill, NY, USA) were used for statistical analyses and to create graphs. Following EACT pretreatment and seizure testing, the GEPR-3s that did not display seizures within the 60 s observation period were considered protected from seizure activity. Therefore, only data obtained in control conditions and following administration of EACT and T16Ainh-A01 were included in the analysis. The incidences of WRSs, GTCSs, and FLE were recorded for each group. The time interval from the start of acoustic stimulus to the onset of the first episode of WRSs was recorded as the seizure latency (or seizure onset). The incidences of WRSs, GTCSs, and FLE were analyzed using Fisher Exact test. The seizure severity was analyzed using the Wilcoxon signed-rank test or Mann Whitney test. The seizure latency was analyzed using one-way ANOVA followed by Bonferroni correction; before performing ANOVA, data were subjected to the Kolmogorov Smirnov test for normality and Levene’s test for homogeneity of variances. The cut-off for statistical significance was *p* < 0.05. Data are presented as percentages (%) for the incidences of WRSs and GTCSs, mean ± S.E.M. for seizure latency, and median score ± median average deviation score for the seizure severity.

## 3. Results

Twenty-four-hour monitoring revealed that administration of EACT at the tested doses did not alter the gross behavior of the GEPR-3s. No loss of righting reflex, Straub tail, sedation, lethargy, ataxia, and spontaneous seizures were observed following EACT and T16Ainh-A01 treatments. In addition, we did not observe an exacerbation of seizure severity (e.g., the occurrence of complete tonic seizures characterized by forelimb and hindlimb extension) in the GEPR-3s subjected to seizure testing. We also did not find notable changes in body temperature.

### 3.1. Effects on EACT at the Dose of 2.5 mg/kg on Acoustically Evoked Seizures in GEPR-3s

**Figure 1:**
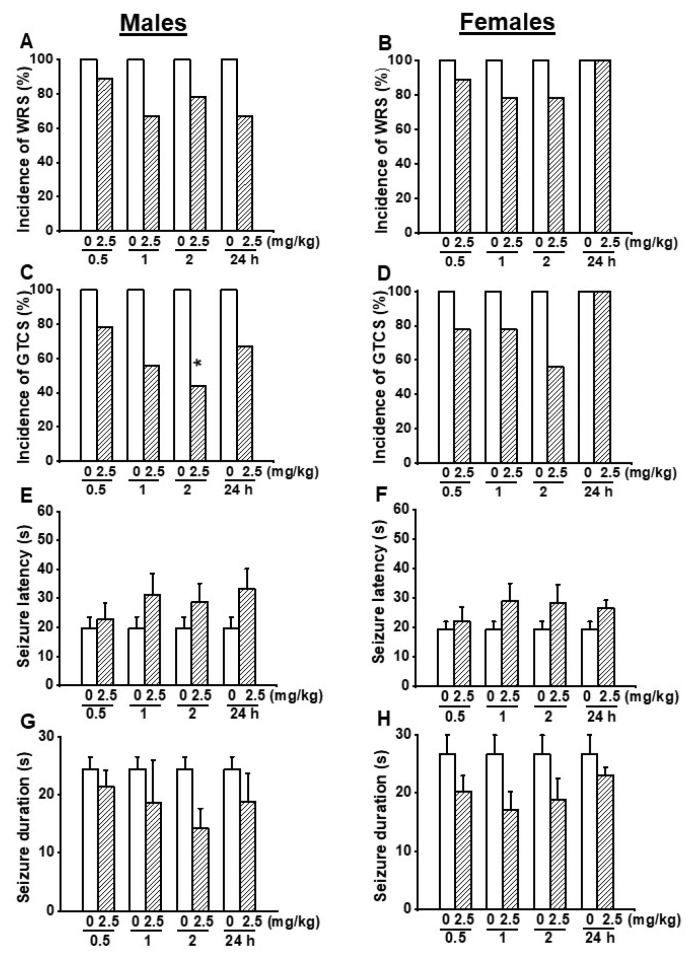
Effects of acute EACT treatment at the dose of 2.5 mg/kg on acoustically evoked seizures. The putative seizure suppressive effects EACT, a potent activator of TMEM16A, were evaluated at different post-treatment time points of 0.5, 1, 2, and 24 h in adult male (*n* = 9) and female (*n* = 9) GEPR-3s. (**A**) EACT treatment did not considerably reduce the incidence of the occurrence of WRSs in males. (**B**) Similarly, EACT treatment also did not considerably reduce the incidence of the occurrence of WRSs in females. (**C**) EACT treatment significantly reduced the incidence of the occurrence of GTCSs at the 2 h post-treatment time point in males. (**D**) However, EACT treatment did not considerably reduce the incidence of the occurrence of GTCSs in females. (**E**) EACT treatment did not considerably delay the seizure latency in males. (**F**) Similarly, EACT treatment also did not considerably delay the seizure onset in females. (**G**) EACT treatment did not considerably reduce the seizure duration in males. (**H**) Similarly, EACT treatment also did not considerably reduce the seizure duration in females. Data from the incidence of the occurrence of WRSs and GTCSs were represented as a mean percentage (%), and the Fisher Exact test was used for analysis. Data from the seizure latency and duration were presented as mean ± S.E.M., and one-way ANOVA followed by Bonferroni correction was used for analysis. Opened and filled bar graphs represent control-treated (pre-EACT) and EACT-treated GEPR-3s, respectively. * *p* < 0.05.

First, we evaluated the effects of acute EACT treatment at the dose of 2.5 mg/kg on acoustically evoked seizure susceptibility in the GEPR-3s. In the control testing conditions (pre-EACT treatment), all-male (*n* = 9) and all-female (*n* = 9) GEPR-3s experienced WRSs (Figure 1A,B), and GTCSs (Figure 1C,D); FLE was observed in one female but not in male GEPR-3s. Fisher Exact test showed that EACT pretreatment did not considerably reduce the incidence of the occurrence of WRSs in male GERP-3s at 0.5, 1, 2, and 24 h post-treatment time points compared with the control testing conditions (Figure 1A). Similarly, EACT also did not considerably reduce the incidence of the occurrence of WRSs in female GEPR-3s at 0.5, 1, 2, and 24 h post-treatment time points compared with the control testing conditions (Figure 1B). The analysis showed that EACT significantly reduced the incidence of the occurrence of GTCSs in male GEPR-3s by 56% (*p* < 0.029) at 2 h but not 0.5, 1, and 24 h post-treatment time points compared with the control testing conditions (Figure 1C). In female GEPR-3s, EACT did not considerably reduce the incidence of the occurrence GTCSs at all tested post-treatment time points compared with the control testing conditions (Figure 1D).

In addition to the incidence of the occurrence of seizures, we also evaluated the effects of EACT’s treatment on the seizure latency and duration (Figure 1E–H). In the control conditions, the seizure latency was 19.78 ± 3.79 s (*n* = 9) and 19.44 ± 2.54 s (*n* = 9) in male and female GEPR-3s, respectively (Figure 1E,F); the seizure duration was 24.44 ± 2.12 s (*n* = 9) and 26.67 ± 3.22 s (*n* = 9) in male and female GEPR-3s, respectively. ANOVA showed that, in male GEPR-3s, EACT did not considerably delay the seizure latency and decreased the seizure duration at all tested post-treatment time points compared with the control testing conditions (Figure 1E,G). Quantification also showed that, in female GEPR-3s, EACT did not considerably delay the seizure latency and decrease the seizure duration at all tested post-treatment time points compared with the control testing conditions (Figure 1F,G).

**Figure 2:**
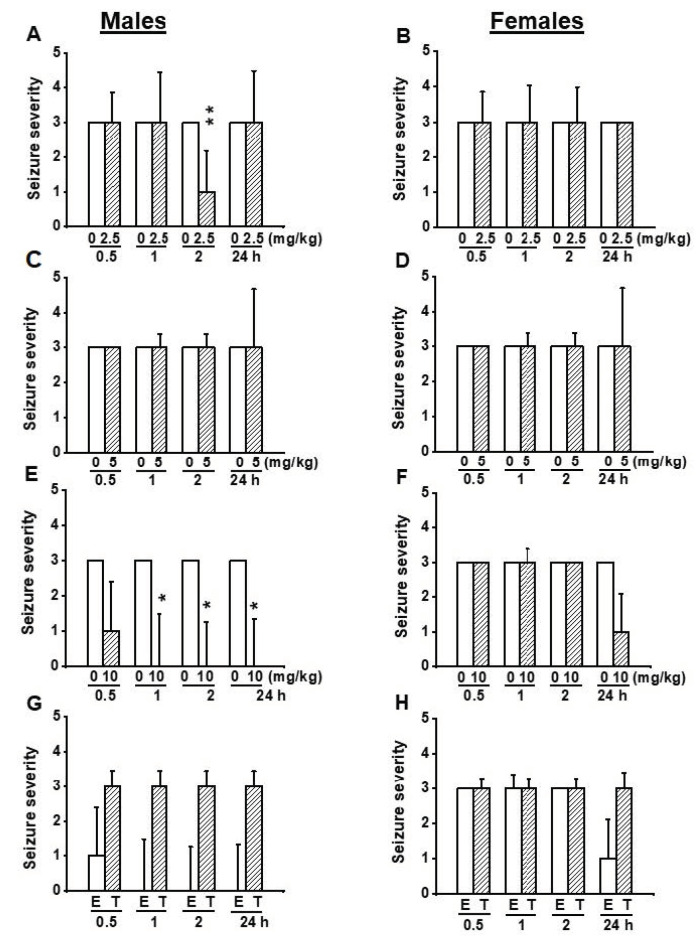
Effects of acute treatment with EACT (E) and T16Ainh-A01 (T), a potent blocker of TMEM16A channels, on the severity of acoustically evoked seizures. The effects of EACT, a potent activator of TMEM16A channels, on the seizure severity were evaluated at various doses (2.5, 5, and 10 mg/kg, p.o.), and at different post-treatment time points (0.5, 1 h, 2 h, and 24 h) in adult male and female GEPR-3s. T16Ainh-A01 was tested only at the dose of 10 mg/kg, p.o.) and the data were compared with EACT (10 mg/kg, p.o.) (**A**) EACT treatment significantly reduced the seizure severity at the 2 h post-treatment time point in males. (**B**) EACT treatment did not alter the seizure severity in females. (**C**) EACT treatment significantly suppressed the seizure severity at 2 and 2 h post-treatment time points in males. (**D**) EACT treatment did not considerably alter the seizure severity in females. (**E**) EACT treatment significantly suppressed the seizure severity at 1, 2, and 24 h post-treatment time points in males. (**F**) EACT treatment did not considerably alter the seizure severity in females. (**G**) In males, T16Ainh-A01 treatment (T) reversed the antiseizure effects following EACT administration (E), but no statistical significance was reached. (**H**) In females, T16Ainh-A01 did alter the effects of EACT. The seizure severity data were represented as median ± median average deviation, and the Wilcoxon signed-rank test was used to compare paired EACT data or T16Ainh-A01 data, and the Mann-Whitney test was used to compare the EACT and T16Ainh-A01 groups. Opened and filled bar graphs represent control-treated (pre-EACT) and EACT treated GEPR-3s (*n* = 9), respectively. * *p* < 0.05, ** *p* < 0.01.

We also examined the effects of EACT on the severity of acoustically evoked seizures. Wilcoxon signed-rank test showed that EACT significantly reduced the seizure severity in male GEPR-3s at 2 h (z = 2.16, *p* < 0.031), but not at 0.5, 1, and 24 h post-treatment time points compared to the control testing conditions (Figure 2A). However, in female GEPR-3s, EACT did not alter the seizure severity at all tested post-treatment time points compared with the control testing conditions (Figure 2B).

### 3.2. Effects on EACT at the Dose of 5 mg/kg on Acoustically Evoked Seizures in GEPR-3s 

**Figure 3:**
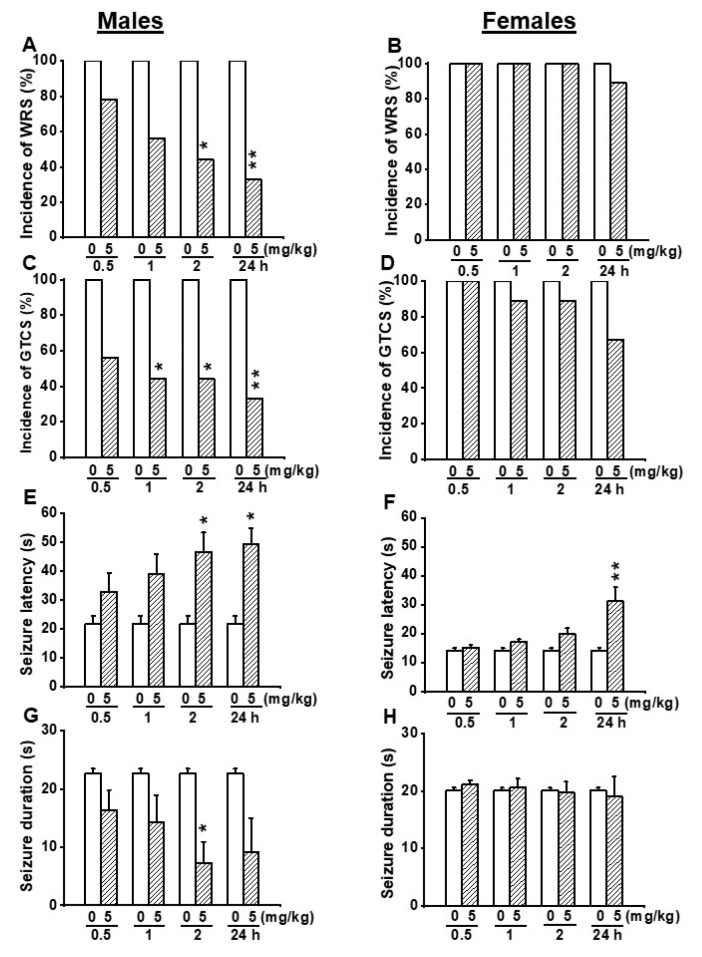
Effects of acute EACT treatment at the dose of 5 mg/kg on acoustically evoked seizures. The effects EACT, a potent activator of TMEM16A, were evaluated at different post-treatment time points of 0.5, 1, 2, and 24 h in adult male (*n* = 9) and female (*n* = 9) GEPR-3s. (**A**) EACT treatment significantly reduced the incidence of the occurrence of WRSs at 2 and 24 h post-treatment time points in males. (**B**) EACT treatment did not considerably reduce the incidence of the occurrence of WRSs in females. (**C**) EACT treatment significantly reduced the incidence of the occurrence of GTCSs at 1, 2, and 24 h post-treatment time points in males. (**D**) EACT treatment did not considerably reduce the incidence of the occurrence of GTCSs in females. (**E**) EACT treatment significantly delayed the seizure onset at 2 and 24 h post-treatment time points in males. (**F**) In females. EACT treatment also significantly delayed the seizure onset at the 24 h post-treatment time point. (**G**) EACT treatment significantly reduced the seizure duration at the 2 h post-treatment time point in males. (**H**) EACT treatment did not alter the seizure duration in females. Data from the incidence of the occurrence of WRSs and GTCSs were represented as a mean percentage (%), and Fisher Exact test was used for analysis. Data from the seizure latency and seizure duration were presented as mean ± S.E.M., and one-way ANOVA followed by Bonferroni correction was used for analysis. Opened and filled bar graphs represent control-treated (pre-EACT) and EACT treated GEPR-3s, respectively. * *p* < 0.05, ** *p* < 0.01.

Next, we evaluated the efficacy of EACT at 5 mg/kg (p.o.) to determine if this dose can either alter the seizure susceptibility in female GEPR-3s or completely suppress seizure susceptibility in male GEPR-3s. In the control testing conditions (pre-EACT treatment), all-male (*n* = 9) and all-female (*n* = 9) GEPR-3s experienced WRSs and GTCSs (Figure 3A–D); FLE was not observed in the GEPR-3s. The Fisher Exact test revealed that, in male GEPR-3s, EACT significantly reduced the incidence of the occurrence of WRSs by 56% (*p* < 0.029) and 67% (*p* < 0.009) at 2 and 24 h post-treatment time points, respectively, compared with control testing conditions (Figure 3A). Furthermore, EACT did not considerably reduce the incidence of the occurrence of WRS at 0.5 h and 2 h post-treatment time points, respectively, compared with control testing conditions (Figure 3A). However, in the females, EACT did not reduce the incidence of the occurrence of WRSs at all tested post-treatment time points compared with the control testing conditions (Figure 3B). Quantification also showed that pretreatment with 5 mg/kg EACT significantly reduced the incidence of the occurrence of GTCSs in male GEPR-3s by 56% (*p* < 0.029), and 56% (*p* < 0.029), and 67% (*p* < 0.002) at 1, 2 and 24 h post-treatment time points, respectively, compared with control testing conditions. However, EACT did not considerably reduce the incidence of the occurrence of WRSs at 0.5 h post-treatment compared to the control testing conditions (Figure 3C). Furthermore, in female GEPR-3s, EACT treatment did not considerably reduce the incidence of the occurrence of GTCSs at all tested post-treatment time points compared with the control testing conditions (Figure 3D).

We also evaluated the effects of EACT’s treatment on seizure latency and duration (Figure 3E–H). In the control conditions, the seizure latency was 21.67 ± 2.93 s (*n* = 9) and 14.33 ± 0.79 s (*n* = 9) in male and female GEPR-3s, respectively (Figure 3E,F); seizure duration was 22.67 ± 0.79 s (*n* = 9) and 20.11 ± 0.48 s (*n* = 9) in male and female GEPR-3s, respectively (Figure 3G,H). ANOVA showed that EACT pretreatment significantly altered the seizure latency in male GEPR-3s (F_(4.40)_ = 3.488, *p* < 0.015). Multiple comparisons revealed that the seizure onset was delayed at 2 h (t = 2.962, *p* < 0.005) and 24 h (t = 3.79, *p* < 0.002) post-treatment time points compared to control testing conditions; the seizure latency was not considerably altered at 0.5 and 1 h post-treatment time points (Figure 3E). The analysis also showed that EACT pretreatment significantly altered the seizure duration. Multiple comparisons revealed that EACT significantly reduced the seizure duration at 2 h (t = 3.075, *p* < 0.038) post-treatment time point compared to control testing conditions; the seizure duration was not considerably reduced at 0.5, 1, and 24 h post-treatment time points (Figure 3G). In female GEPR-3s, ANOVA showed that EACT pretreatment significantly alters the seizure latency (F_(4.40)_ = 7.846, *p* < 0.00009). Multiple comparisons revealed that EACT significantly delayed the seizure onset at 24 h (t = 4.895, *p* < 0.002) post-treatment time points compared with control testing conditions; the seizure latency was not considerably increased at 0.5, 1, and 2 h post-treatment time points (Figure 3F). The analysis also showed that EACT treatment did not alter the seizure duration in females GEPR-3s (Figure 1H).

We also evaluated the effects of EACT pretreatment on the severity of acoustically evoked seizures. Wilcoxon signed-rank test showed that at a dose of 5mg/kg, EACT treatment significantly suppressed the seizure severity in male GEPR-3s at 2 h (z = 2.22, *p* < 0.033), and 24 h (z = 2.33, *p* < 0.031) post-treatment time points compared with the control testing conditions; the seizure severity was not considerably reduced or changed at 1 and 0.5 h post-treatment, respectively (Figure 2C). In female GEPR-3s, EACT did not alter the seizure severity compared with the control testing conditions (Figure 2D).

### 3.3. Effects on EACT at the Dose of 10 mg/kg on Acoustically Evoked Seizures in GEPR-3s

**Figure 4:**
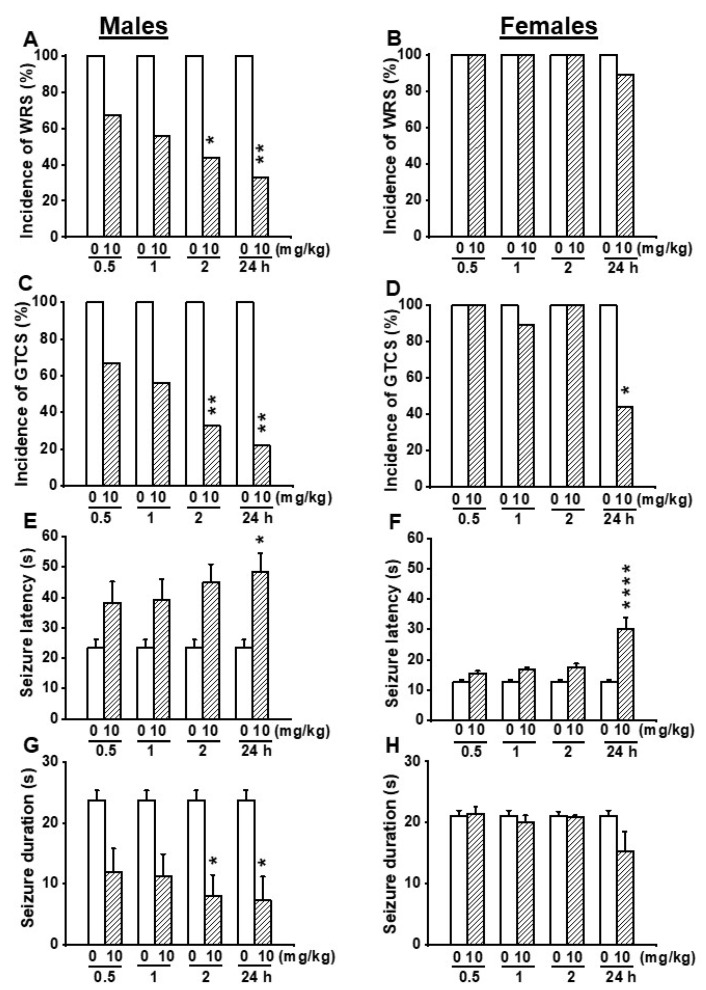
Effects of acute EACT treatment at the dose of 10 mg/kg on acoustically evoked seizures. The effects of EACT, a potent activator of TMEM16A, were evaluated at different post-treatment time points of 0.5, 1 h, 2 h, and 24 h in adult male and female GEPR-3s. (**A**) EACT treatment significantly reduced the incidence of the occurrence of WRSs at 2 and 24 h time points in males. (**B**) However, EACT treatment did not considerably alter the incidence of the occurrence of WRSs in females. (**C**) EACT treatment significantly reduced the incidence of the occurrence of GTCSs at 2 and 24 h post-treatment time points in males. (**D**) EACT treatment also significantly reduced the incidence of the occurrence of GTCSs at 24 h post-treatment time point in females. (**E**) EACT treatment significantly delayed the seizure onset at 24 h post-treatment time points in males. (**F**) Similarly, EACT treatment also significantly delayed the seizure onset at 24 h post-treatment time point in females. (**G**) EACT treatment significantly reduced the seizure duration at 2 and 24 h post-treatment time points in males. (**H**) However, in females, EACT did not considerably alter the seizure duration. Data from the incidence of WRSs and GTCSs were represented as a mean percentage (%), and Fisher Exact test was used for analysis. Data from the seizure latency and seizure duration were presented as mean ± S.E.M., and one-way ANOVA followed by Bonferroni correction was used for analysis. Opened and filled bar graphs represent control-treated (pre-EACT) and EACT treated GEPR-3s (*n* = 9), respectively. * *p* < 0.05, ** *p* < 0.01, **** *p* < 0.0001.

Since no anticonvulsant effects were seen in female GEPR-3s following administration of EACT at the doses of 2.5 and 5 mg/kg, we evaluated the effects of EACT pretreatment at the dose of 10 mg/kg on acoustically evoked seizure susceptibility in the GEPR-3s. In the control testing conditions (pre-EACT treatment), all-male (*n* = 9) and all-female (*n* = 9) GEPR-3s experienced WRSs and GTCSs (Figure 4A–D). FLE was not observed in both male and female GEPR-3s. The Fisher Exact test showed that the incidence of the occurrence of WRSs, in male GEPR-3s, was significantly reduced by 56% (*p* < 0.029) and 67% (*p* < 0.009) at 2 and 24 h post-treatment time points compared with control testing conditions; no considerable reduction of the incidence of the occurrence of WRSs was seen at 0.5 and 1 h post-treatment time points (Figure 4A). However, in females, EACT did not considerably alter the incidence of the occurrence of WRSs at all tested post-treatment time points compared with the control testing conditions (Figure 4B). We also evaluated the effects of EACT on the incidence of the occurrence of GTCSs. Analysis revealed that EACT significantly reduced the incidence of the occurrence of GTCSs, in male GEPR-3s, by 67% (*p* < 0.009) and 78% (*p* < 0.002) at 2 and 24 h post-treatment time points compared with control testing conditions; no considerable reduction of the incidence of the occurrence of GTCSs was seen at 0.5 h and 1 h post-treatment time points (Figure 4C). In female GEPR-3s, EACT significantly reduced the incidence of the occurrence of GTCSs by 56% (*p* < 0.029) at 24 h post-treatment time point compared with the control testing conditions; no considerable change was observed at other post-treatment time points (Figure 4D).

We also evaluated the effects of EACT pretreatment on the severity of acoustically evoked seizures. The Wilcoxon signed-rank test showed that EACT significantly reduced the seizure severity in male GEPR-3s at 1 h (z = 2.22, *p* < 0.031), 2 h (z = 2.22, *p* < 0.031), and 24 h (z = 2.33, *p* < 0.019) post-treatment time points compared with control testing conditions. However, the seizure severity was not considerably decreased at the 0.5 h post-treatment time point (Figure 2E). In females, EACT did not considerably alter the seizure severity up to 2 h post-treatment time points compared to the control conditions (Figure 2F).

We also quantified the effects of EACT pretreatment on seizure latency and seizure duration. In the control conditions, the seizure latency was 23.56 ± 2.49 s (*n* = 9) and 12.78 ± 0.68 s (*n* = 9) in male and female GEPR-3s, respectively (Figure 4E,F); the seizure duration was 23.67 ± 1.67 s (*n* = 9) and 21.11 ± 0.90 s (*n* = 9) in male and female GEPR-3s, respectively (Figure 4G,H). ANOVA showed that EACT pretreatment significantly alter the seizure latency in male GEPR-3s (F_(4.40_ = 2.786, *p* < 0.0393). Multiple comparisons revealed that EACT significantly delayed the seizure onset at the 24 h (t = 3.024, *p* < 0.043) post-treatment time point in male GEPR-3s compared with control testing conditions. No considerable delay of the seizure onset was seen at the other tested post-treatment time points (Figure 4E,F). In female GEPR-3s, the analysis showed that EACT pretreatment significantly alter the seizure latency (F_(4.40)_ = 12.159, *p* < 0.000001). Multiple comparisons revealed that the seizure onset was only significantly delayed at the 24 h (t = 6.351, *p* < 0.0001) post-treatment time point compared with the control testing conditions. EACT did not considerably alter the seizure latency at the other tested post-treatment time points (Figure 4F). We also evaluated the effects of EACT pretreatment on the seizure duration. Analysis also showed that EACT pretreatment significantly alters the seizure duration in male GEPR-3s (F_(4.40)_ = 3.776, *p* < 0.0107). Multiple comparisons revealed that, in male GEPR-3s, EACT significantly reduced the seizure duration at 2 h (t = 3.254, *p* < 0.0232) and 24 h (t = 3.417, *p* < 0.0147) post-treatment time points compared with control testing conditions. No considerable changes in the seizure duration were seen at other post-treatment time points (Figure 4G). However, EACT did not considerably alter the seizure duration in female GEPR-3s (Figure 4H).

**Figure 5:**
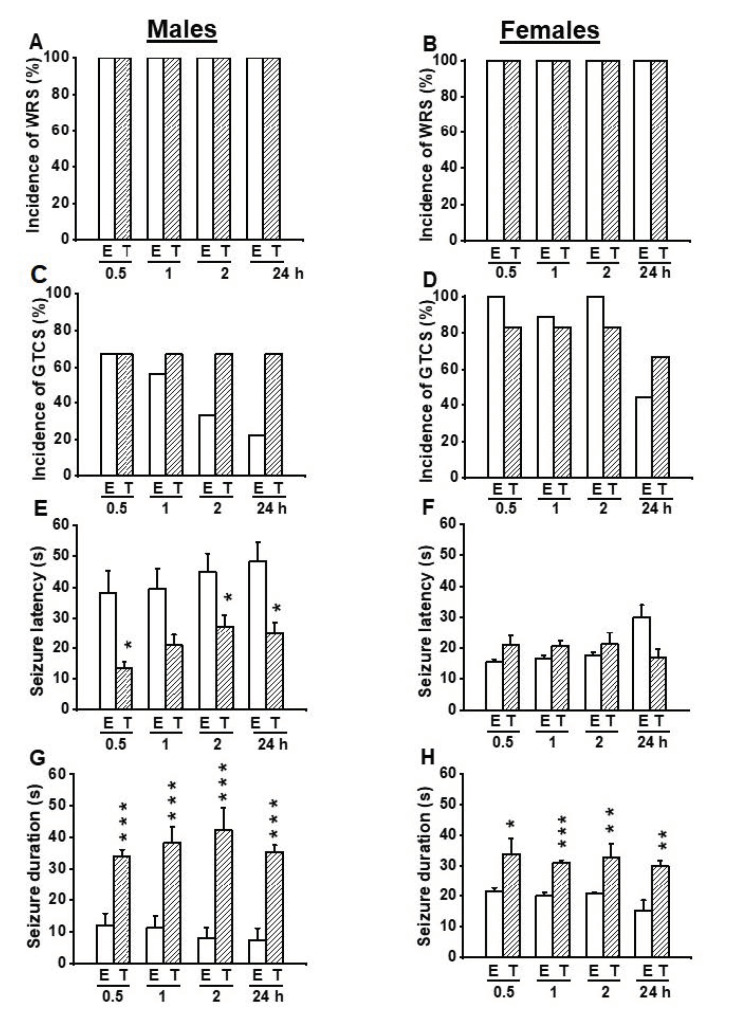
Effects of acute treatment with EACT (E) and T16Ainh-A01 (T), a potent activator and blocker of TMEM16A channels, respectively. The effects of EACT (*n* = 9) and T16Ainh-A01 (*n* = 6) on acoustically evoked seizures were evaluated at the dose of 10 mg/kg and at various posttreatment time points (0.5, 1 h, 2 h, and 24 h) in adult male and female GEPR-3s. Data were compared to determine the extent to which T16Ain-A01 treatment reverses the antiseizure effects seen in the EACT-treated group. (**A**) The incidence of the occurrence of WRSs was similar in both the EACT-treated and the T16Ainh-A01-treated groups in males. (**B**) Likewise, the incidence of the occurrence of WRSs was similar in both the EACT-treated group and T16Ainh-A01-treated group in females. (**C**) T16Ainh-A01 treatment reversed the reduced incidence of GTCSs seen in the EACT-treated group in males, but this effect did not reach statistical significance. (**D**) Similarly, T16Ainh-A01 treatment did alter the effects of EACT on the incidence of the occurrence of WRSs in female GEPR-3s. (**E**) T16Ainh-A01 treatment significantly reversed the effect of EACT treatment on the seizure latency in males. (**F**) In females, T16Ainh-01 also significantly reversed the effect of EACT on the seizure latency. (**G**) T16Ainh-A01 treatment significantly reversed the effect of EACT treatment on the seizure duration in males. (**H**) In females, T16Ainh-01 also significantly reversed the effect of EACT on the seizure duration. Data from the incidence of the occurrence of WRSs and GTCSs were represented as a mean percentage (%), and Fisher Exact test was used for analysis. Data from the seizure latency and duration were presented as mean ± S.E.M., and one-way ANOVA followed by Bonferroni correction was used for analysis. Opened and filled bar graphs represent EACT-treated GEPR-3s (E) and T16Ainh-A01-treated GEPR-3s (T). * *p* < 0.05, ** *p* < 0.01, *** *p* < 0.001.

In another set of experiments, we probed the extent to which inhibition of TMEM16A channels reversed the antiseizure effects seen following activation of these channels. Thus, we evaluated the effects of T16Ainh-A01 (10 mg/kg, body weight, p.o.), a potent inhibitor of TMEM16A channels on the acoustically evoked seizure susceptibility in both male (*n* = 6) and female (*n* = 6) GEPR-3s. Quantification showed that T16Ainh-A01 pretreatment did not alter the incidence of the occurrence of WRSs and GTCSs, the seizure latency, the seizure duration, and the seizure severity (compare Figure 4 and Figure 5, and see Figure 2E–H). Comparison of the effects of EACT and T16Ainh-A01 on the seizure susceptibility revealed T16Ainh-A01 significantly rescued EACT-induced delay of the seizure latency (Figure 5E) and EACT-induced decreases in the seizure duration (Figure 5H) in male GEPR-3s. In female GEPR-3s, T16Ainh-A01 significantly rescued EACT-induced increases in the seizure latency 24-h post-treatment (Figure 5F). T16Ainh-A01 also increased the seizure duration compared to EACT pretreatment in female GEPR-3s at all tested post-treatment time points (Figure 5H). However, both EACT and T16Ainh-A01 did not alter the incidence of the occurrence of WRSs in both male and female GEPR-3s (Figure 5A,B). T16Ainh-A01 also did not considerably rescue the suppressive effects of EACT on the incidence of the occurrence GTCSs (Figure 5C,D) and the seizure severity (Figure 2G,H) in both male and female GEPR-3s.

Finally, we evaluated the effects of seizure susceptibility between male and female GEPR-3s used in this study. Analysis revealed no considerable changes in the seizure latency, seizure severity, incidence of the occurrence of WRSs and GTCSs, and seizure severity between male and female GEPR-3s (data not shown).

## 4. Discussion

In this study, we evaluated the role of activating TMEM16A channels as a putative novel mechanism for seizure suppression in the GEPR-3s. We found that activation of TMEM16A channels reduced the occurrence of both WRSs and GTCSs, and the seizure severity. We also found that activation of TMEM16A channels delayed the seizure onset and reduced the seizure duration. These antiseizure effects were primarily seen in male GEPR-3s. The effects on the seizure latency and duration (and seizure severity to a lesser extent) were markedly reversed by inhibiting TMEM16A channels. Together, these findings suggest that TMEM1A channels may play essential roles in the pathophysiology of inherited seizure susceptibility in the GEPR-3s. The prolonged seizure onset following activation of TMEM16A channels suggests that these channels may play a role in the propagation of seizure activity from the initiation site to networks responsible for the expression of seizure phenotypes. The reduced seizure duration following activation of TMEM16A channels indicates that these channels may play a role in the mechanisms underlying seizure termination. Finally, the suppression of the seizure severity suggests a role of TMEM16A channels on the seizure threshold and seizure initiation.

Acoustically evoked seizure susceptibility is associated with increased VGCC currents in IC neurons, suggestive of massive Ca^2+^ influx resulting in abnormal levels of intracellular Ca^2+^ that can activate chloride channels among other Ca^2+^-dependent mechanisms. Therefore, CaCCs may play a role in the mechanisms underlying neuronal hyperexcitability that leads to seizures. Accordingly, selective deletion of TMEM16C channels in the brain exhibited enhanced susceptibility to hyperthermia-induced tonic-clonic seizures and decreased the seizure latency in rodent pups [30]. Furthermore, knockdown of TMEM16B channels in thalamocortical neurons reduced spike frequency adaptation and significantly decreased the afterhyperpolarization conductance, consistent with the enhanced neuronal excitability that can lead to seizures [31]. Interestingly, reduced spike frequency adaptation and slow afterhyperpolarization conductance were found in CA1 and CA3 neurons of the hippocampus in the GEPR-9, the most severe seizure severity strain of the GEPRs [32,33]. Such altered spike frequency adaptation and slow afterhyperpolarization may also occur in the GEPR-3s and contribute to enhancing the seizure susceptibility [14,34]. The mechanisms of how activation of CaCCs suppresses seizures are complex. Nevertheless, we posit that activation of CaCCs allows chloride influx that contributes to hyperpolarization of the neurons and modulates the spike-frequency adaptation via the shunting effect, leading to seizure suppression in the GEPR-3s [35,36,37,38]. Activation of CaCCs also contributes to elevated intraneuronal chloride levels, which have been reported to accompany seizure activity [18,19,39]. However, elevated intraneuronal chloride loading by itself does not trigger a complete ictal activity [39].

Other molecular targets can mediate the antiseizure effects of EACT. Evidence indicates that EACT also activates the transient receptor potential vanilloid 1 (TRPV1) channel in addition to CaCCs, suggesting a potential role of these channels in EACT’s antiseizure effects [40]. However, we previously reported that capsazepine, a potent inhibitor of TRPV1 channels, completely suppressed the seizure susceptibility in female (but not male, as seen in the present study with EACT) GEPR-3s [41]. These findings ruled out the possible role of TRPV1 channels in EACT’s antiseizure effects in male GEPR-3s. In the present study, we also found that EACT’s antiseizure effects were mainly seen in male but not female GEPR-3s, suggesting that male GEPR-3s are more sensitive to the activation of CaCCs. The underlying biological mechanisms for the differential sex effect of EACT’s antiseizure effects are unknown in GEPR-3s. Nevertheless, sex-related EACT’s antiseizure effects may be due to sex differences in inherited seizure susceptibility in GEPR-3s. However, this study found no notable differences in the parameters and phenotypes of acoustically evoked seizures between male and female GEPR-3s. Steroid hormones and endogenous neuro-steroids have been implicated as factors contributing to sex differences in epilepsy and may play a role in the lack of EACT’s antiseizure effects in female GEPR-3s [42]. However, female GEPR-3s consistently exhibited seizures over 30 days of repetitive acoustic stimulations, ruling out the possible role of steroids and neuro-steroids in EACT’s antiseizure effects [43]. A potential sex-related mechanism of the EACT’s antiseizure effects may include altered function and expression of TMEM16A channels, at least in the IC, the initiation site of acoustically evoked seizures in the GEPR.

Despite the novelty of our findings, this study has a limitation of its translational value because no mutations of the genes encoding for CaCCs are yet associated with seizure phenotypes and epilepsy syndromes in humans [2,44].

In conclusion, activation of TMEM16A channels is sufficient to suppress seizure susceptibility in the male GEPR-3s, providing a putative novel cellular mechanism for controlling tonic-clonic seizures and epilepsy.

## Data Availability

Data are available upon request from the corresponding author.
